# Supplementary material for: Selective single molecule sequencing and assembly of a human Y chromosome of African origin
Source: Nat Commun. 2019 Jan 2;10:4. doi: 10.1038/s41467-018-07885-5 (PMC6315018; doi:10.1038/s41467-018-07885-5)
Supplement: Supplementary file 1 — Supplementary Information [file 41467_2018_7885_MOESM1_ESM.pdf]

Supplementary Material for:

Selective single molecule sequencing and assembly of a human Y chromosome of African origin

Kuderna, Lizano et al. , 2018

Supplementary Notes

Supplementary Note 1: Residual error rate estimate

To estimate the residual error rate within our assembly, we identified regions aligning to the X-degenerate and the X-transposed regions in GRCh38, that together comprise a callable space of 11,985,955 bp. We then checked the variant calls derived from the Illumina data against our reference. To this end we mapped all trimmed data onto the assembly and called single nucleotide variants and small insertions and deletions and extracted all regions identified as single copy homologs in GRCh38 (see Methods).

We estimated error rate in two ways: First, we only checked single nucleotide changes. We call 2,043, and thus estimate the remaining base error rate to be 0.00005 $^{\text{-bp.}}$

Secondly, as most remaining errors within the Nanopore data are deletions, we calculated the size difference between the allele calls +1 as the base for residual errors. By this means, we calculate the remaining base error rate to be of 0.0002 $^{\text{-bp}}$.

Supplementary Note 2: Enrichment specificity

The enrichment specificity was defined as follows: We calculated the length of each chromosome in GRCh38 and subtracted the number of gapped bases within it. For the autosomes, the resulting number was multiplied by two to account for ploidy, and all weights were normalized by the total length of the assembly (accounting for ploidy). We then calculated the observed coverage on each chromosome from either Nanopore or Illumina data, and normalized it by the total number of

mapped bases. This resulting 'real' weight was then divided by the theoretical weight of equal drawing mentioned above to calculate the enrichment specificity.

Supplementary Note 3: Genotype concordance to 1000 genomes calls

We compared the assembly with reported single nucleotide variants (SNVs) for the same individual in the 1000 Genomes project1. We retrieved alternative alleles for the assembly using maffilter2, and performed a liftover of coordinates from hg19 to hg38 for SNVs from the 1000 Genomes data using rtracklayer3 and GenomicRanges4

We find a total of 2,693 SNVs where the assembly differs from the reference genome and data from Illumina sequencing from the 1000 Genomes data is available. Of these, 2,683 (99.6%) show the same alternative allele, eight variants show the reference allele for this individual in the 1000 Genomes data, and two variants show a different alternative allele. Half of the discordant cases are singletons or doubletons across the 1000 Genomes data, and might represent sequencing errors, while for the other cases the assembly strategy might improve the genotyping of the Y chromosome.

Overall, the concordance with previously reported SNVs is high. However, we find an additional 21,350 positions with alternative alleles in the new assembly, some of which might have not been observed due to an extensive filtering and liftover between reference genomes.

We used the available short-read sequences of this individual, performed a new mapping against the same reference genome (hg38) using bwa mem (0.7.7)5 and standard quality filtering (duplicate removal, indel realignment) using samtools (1.6)6 and GATK (3.7,)7. We called the genotypes using GATK UnifiedGenotyper, and used generous cutoffs for comparison (coverage between 2-fold and 75-fold, mapping quality larger than 20, less than 10% of reads with MQ0, and

excluding heterozygous genotypes). Here, we observe 3,451 SNVs where genotypes called both from the assembly and the Illumina sequencing show the same alternative allele. Six positions (0.17% of alternative SNVs) show different alternative alleles between the two approaches. However, we also find 5,187 positions where the assembly shows an alternative allele, but the short-read sequencing supports the reference allele. These might be sequencing errors in the long read-based assembly.

The public data on this individual is of relatively low coverage (~5-fold for the haploid Y chromosome). In order to increase confidence on these observations, we mapped short-read sequences from deeper sequencing (~60-fold coverage) of the Y chromosome after chromosome sorting, and called genotypes as described above, with the only difference that a haploid genotype call was performed. We used sites with a coverage between 3-fold and 99-fold and the same filters as above. Here, we find 4,263 concordant alternative genotypes, where the alternative genotype from short-read sequencing data and the long-read based assembly are identical. However, we also find 6,635 discordant alternative genotypes, where the short-read sequencing data supports the reference allele, while the assembly differs.

In both cases, a larger number of discordant sites falls in heterochromatic, ampliconic and other regions with low confidence, hence are not reliable genotypes in any case. However, 35.6% (1,389) of alternative alleles in the X-degenerate region obtained from the assembly are discordant with short-read sequencing data and might not be reliable genotypes. This is most likely due to high error rates in long-read Nanopore sequencing.

Supplementary Figures

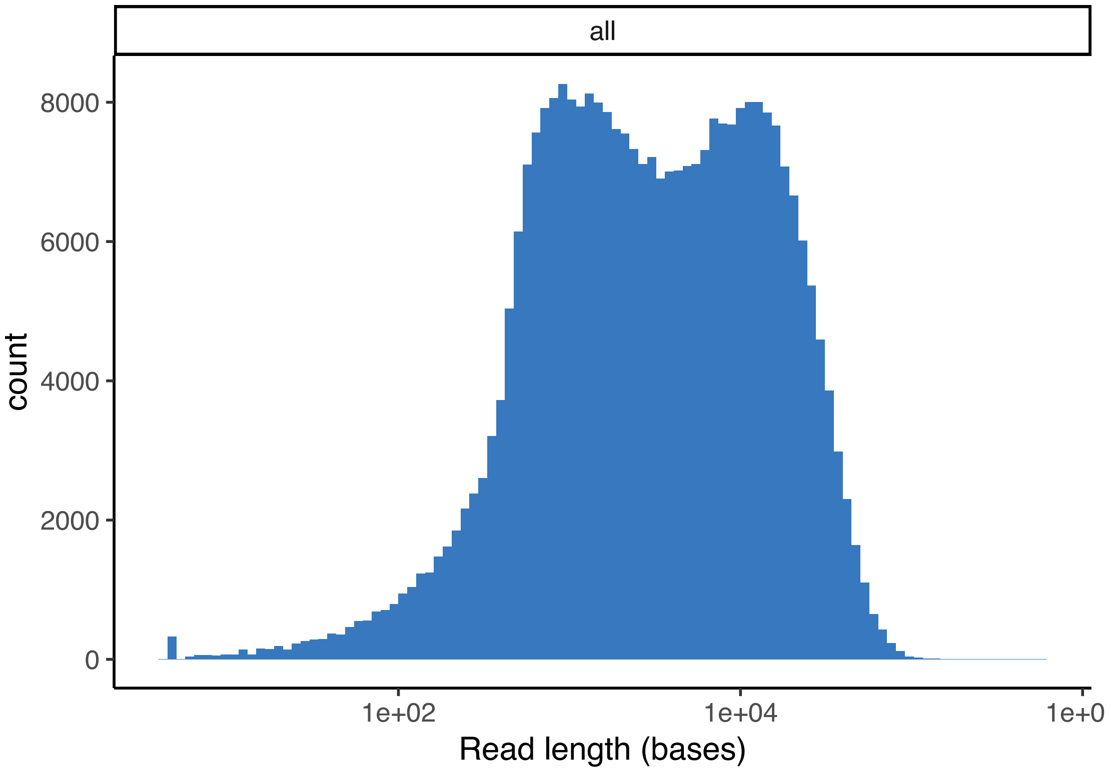

Supplementary Figure 1

Combined read length distribution of all four runs

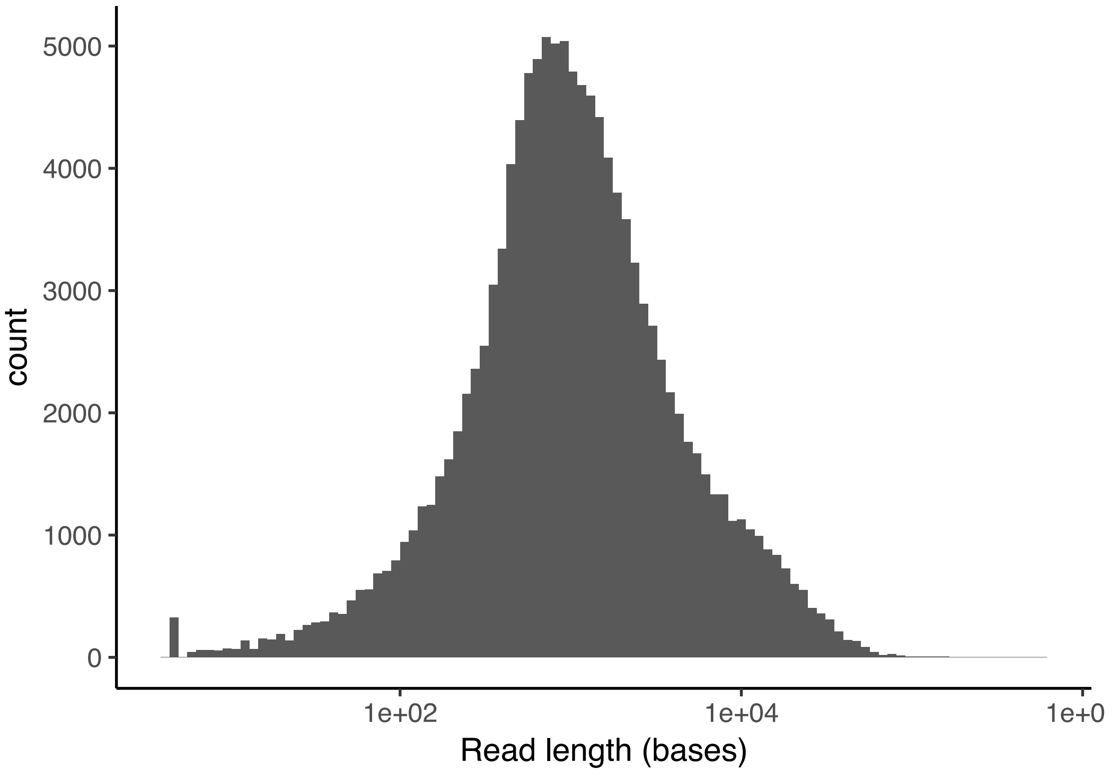

Supplementary Figure 2

Read-length distribution of reads that did not map onto GRCh38

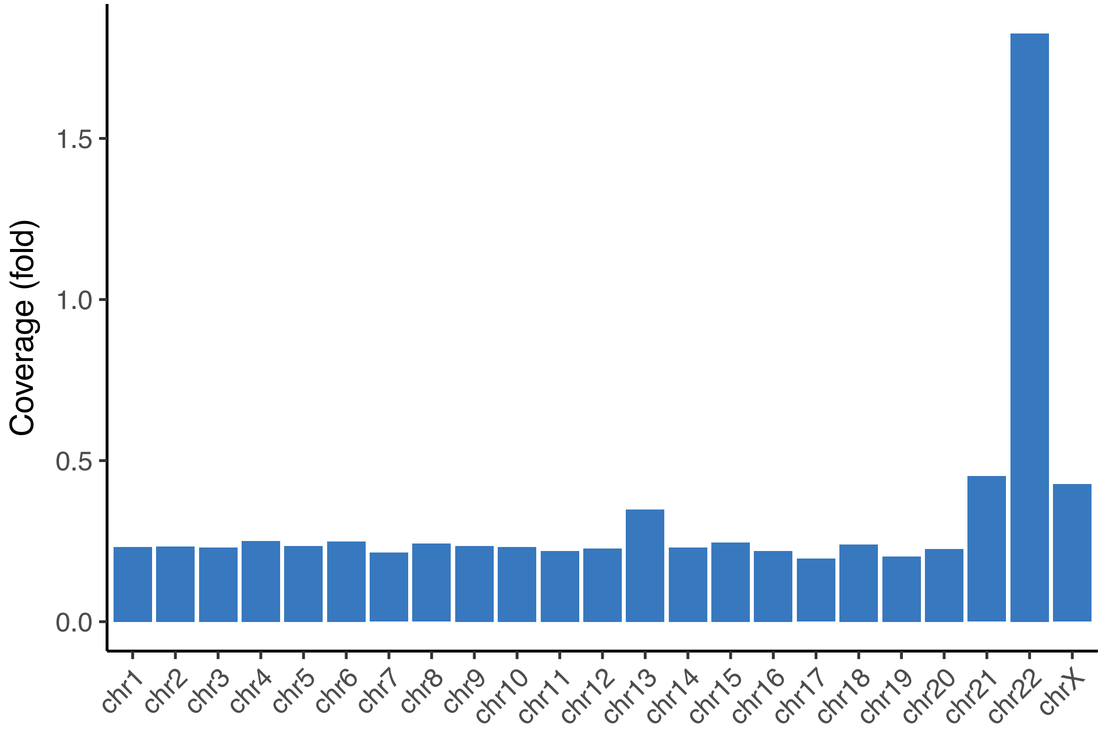

Supplementary Figure 3

Sequencing coverage of Nanopore data on GRCh38 without Y chromosome for scale. The coverage on the Y chromosome is 31X.

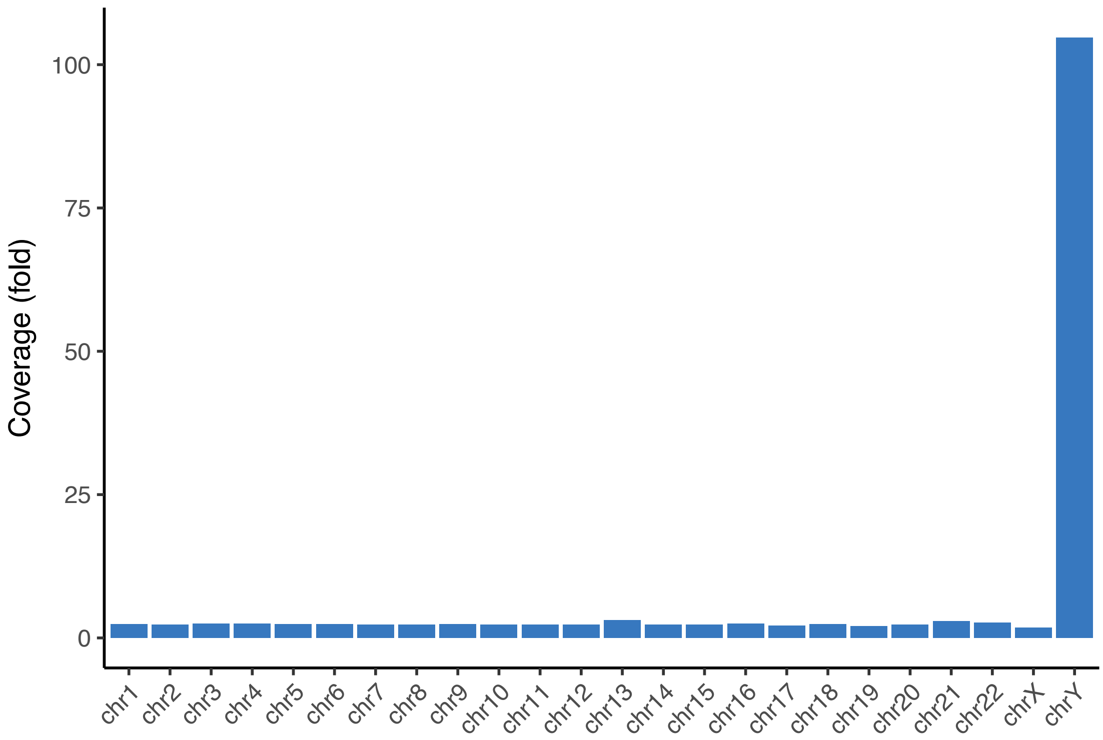

Supplementary Figure 4

Sequencing coverage of Illumina data on GRCh38, MQ >0. The enrichment specificity on the Y chromosomes is slightly lower than in the Nanopore data, presumably due to random fluctuations in template selection upon PCR-amplification.

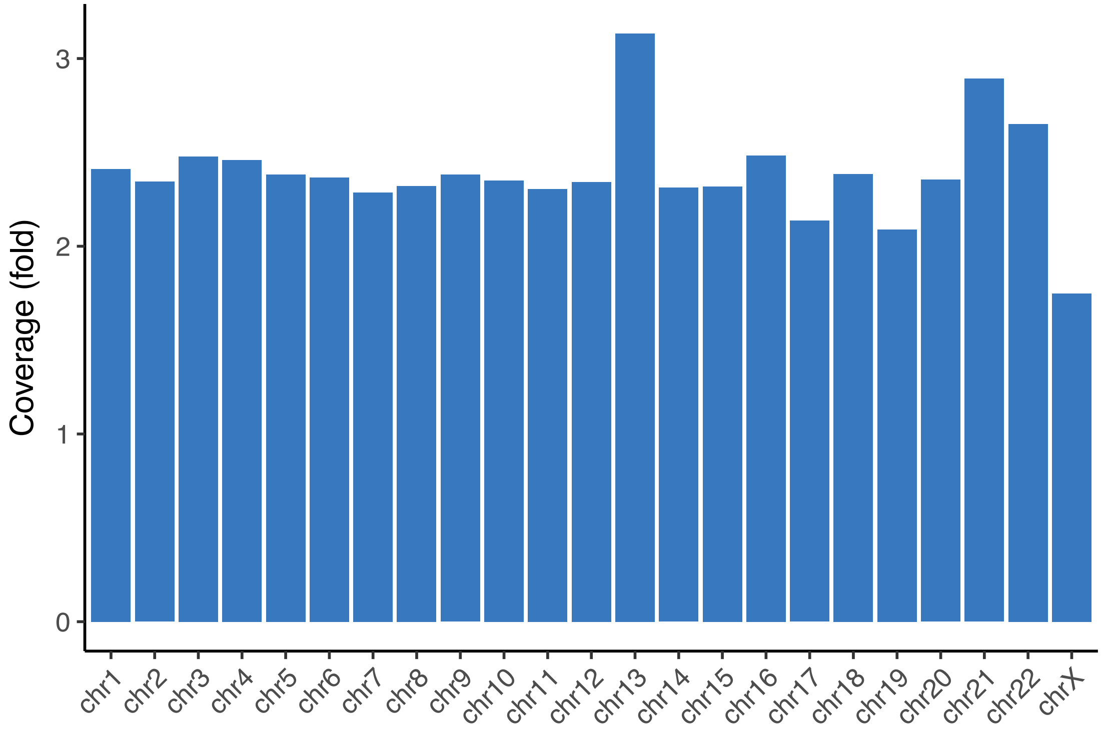

Supplementary Figure 5

Sequencing quality of Illumina data on GRCh38 without Y chromosome, MQ>0

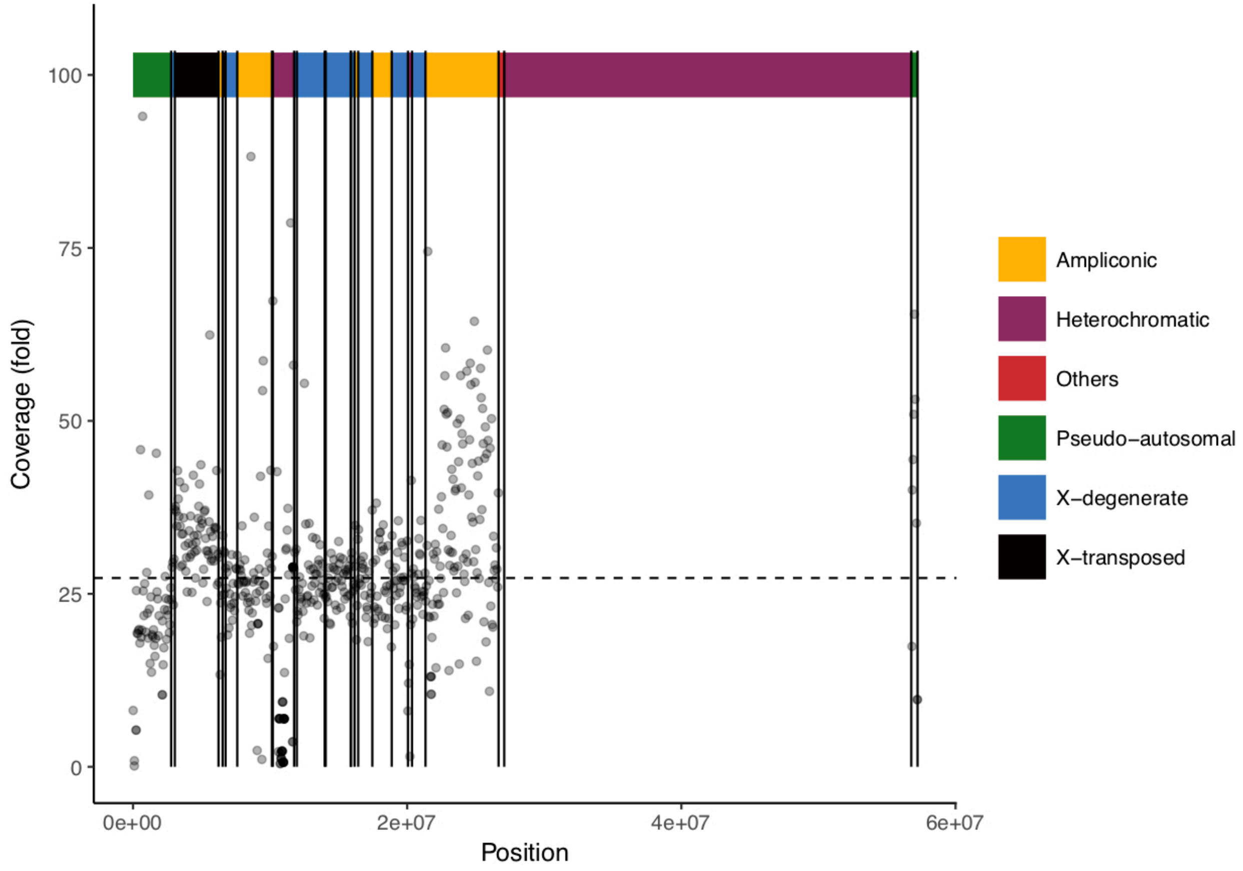

Supplementary Figure 6

Sequencing coverage of Nanopore data in bins of 50 Kb data along the Y chromosome, including information on the discrete sequence classes. A steep drop-off in coverage coinciding with the PAR-1 boundary can be observed. Variable read depths for the Ampliconic and X-transposed regions are likely due to multimappings of reads inflating the coverage. We hypothesize the drop off to be due unfinished recombination events upon arresting the chromosomes in metaphase. The broken PAR-1 pieces would not sort with the main cluster of Y chromosomes anymore, and thus be absent from the sequencing data.

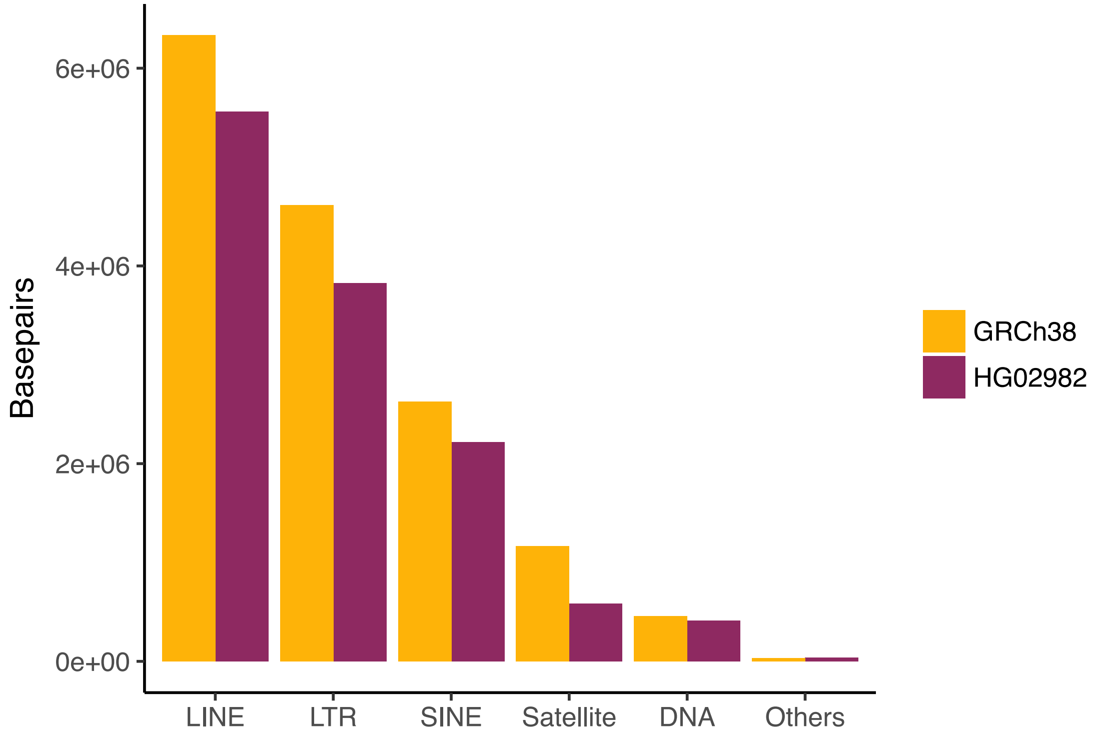

Supplementary Figure 7

Repeat content of major interspersed repeats between the two assemblies. The relative amount of repeats in each family is comparable between the two assemblies, with the exception of satellite sequences that are underrepresented in HG02982.

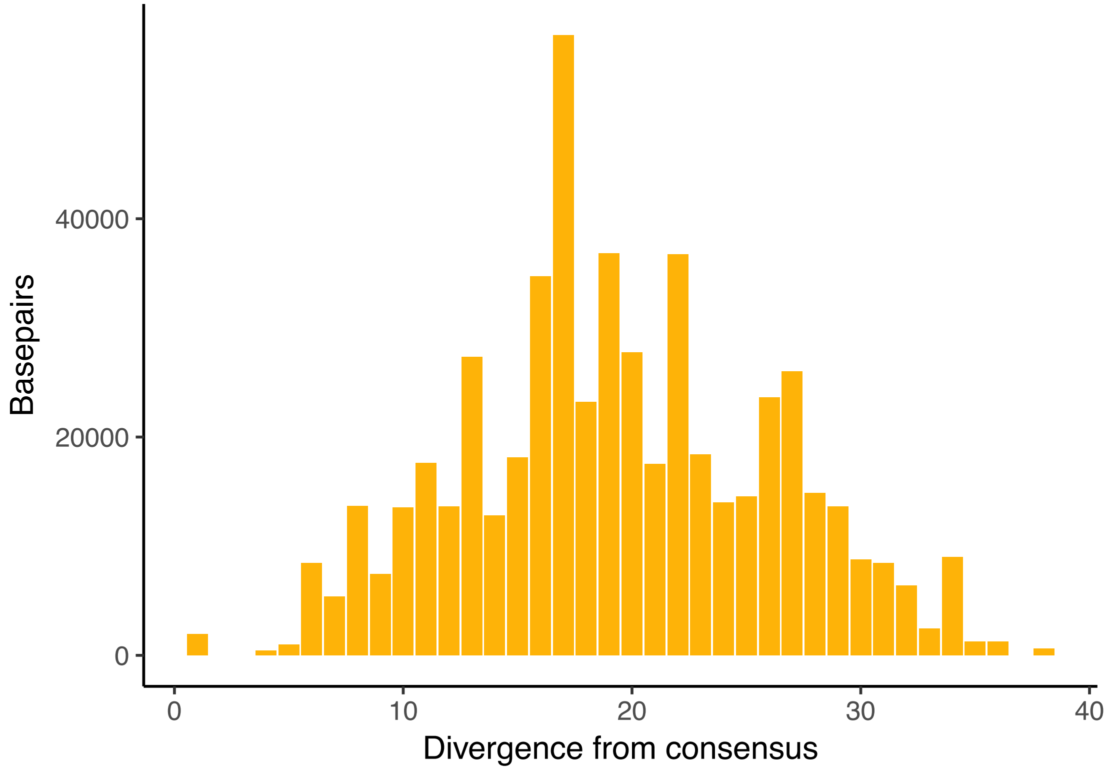

Supplementary Figure 8

Repeat landscape of LTR elements in GRCh38 which are not covered by alignments in HG02982. These elements amount to 538634 bases, and are predominantly found in ampliconic regions, which are not completely resolved in HG02982.

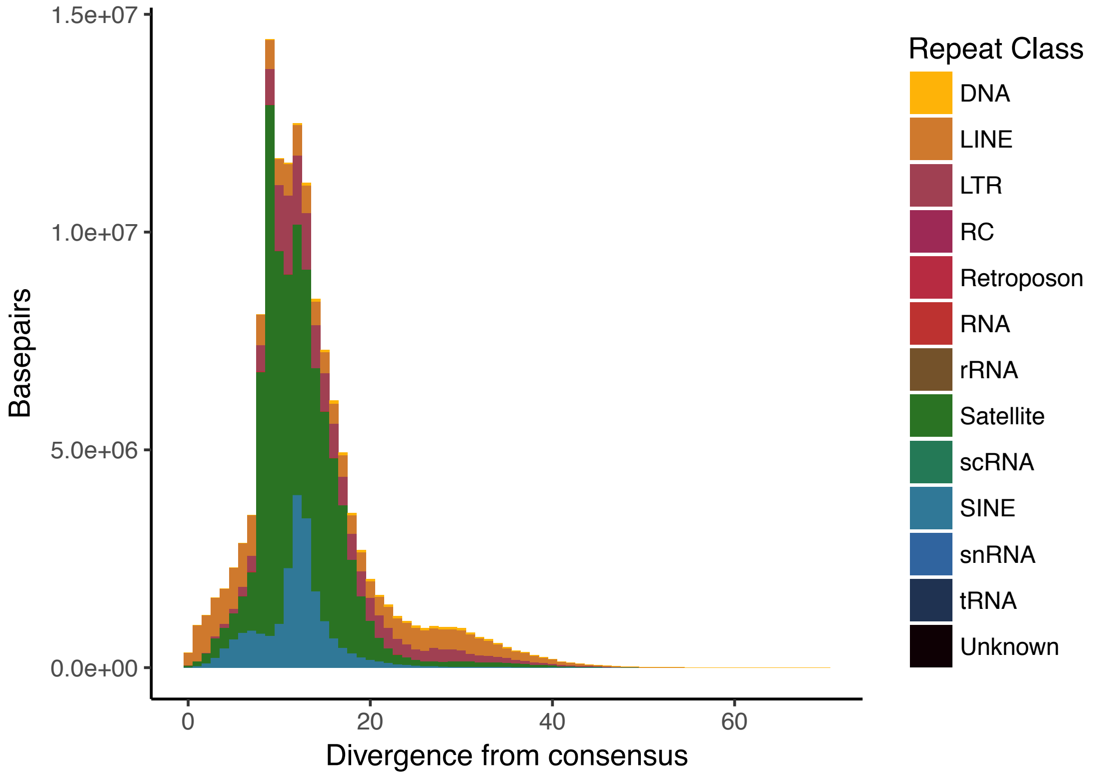

Supplementary Figure 9

Repeat content of unassembled data. Satellite sequences are the main sequence class within poorly assembled regions. We find 34 contigs amounting to 808 kb that were filtered out because of low coverage or repeat sequences. Satellite sequences have been found to be disproportionally represented in regions that are challenging to reconstruct. Accordingly, we find them to amount to 16% of all sequences that did not properly assemble, and therefore to be 6-fold enriched within these data. Additionally, there are around 356 Mbps of single, unassembled reads (not represented within the figure above).

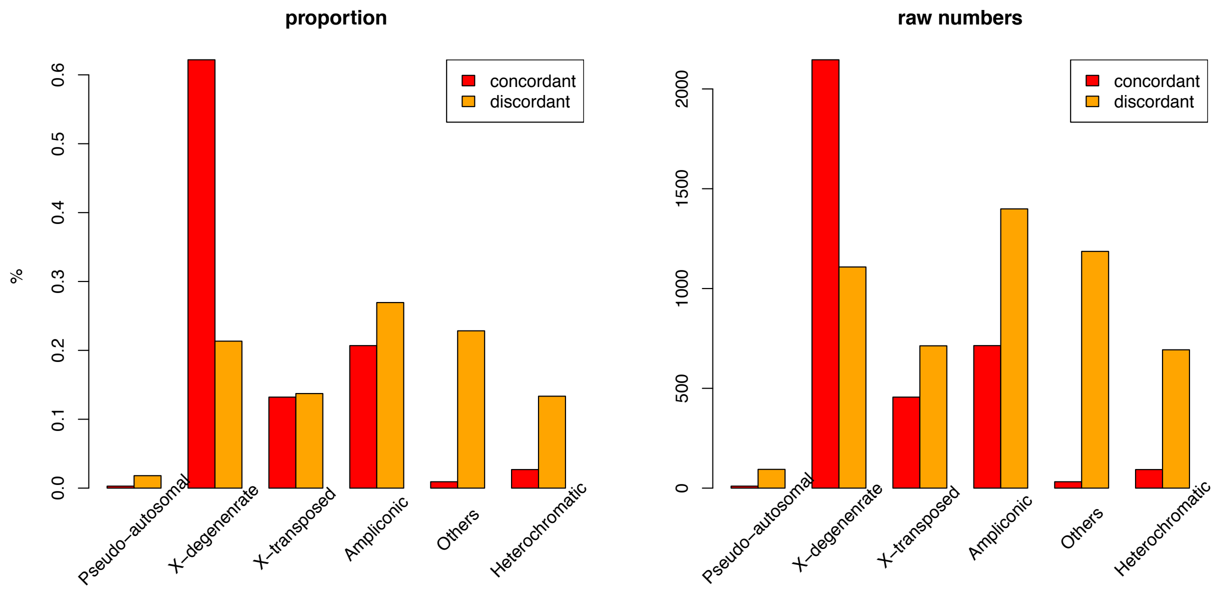

Supplementary Figure 10

Proportion and number of concordant and discordant genotypes as defined in the supplementary section ‘Genotype concordance to 1000 genomes calls’ using the Y chromosomal sequences from the publicly available sequence data from the 1000 genomes project (Poznik et al), stratified by their respective sequence classes.

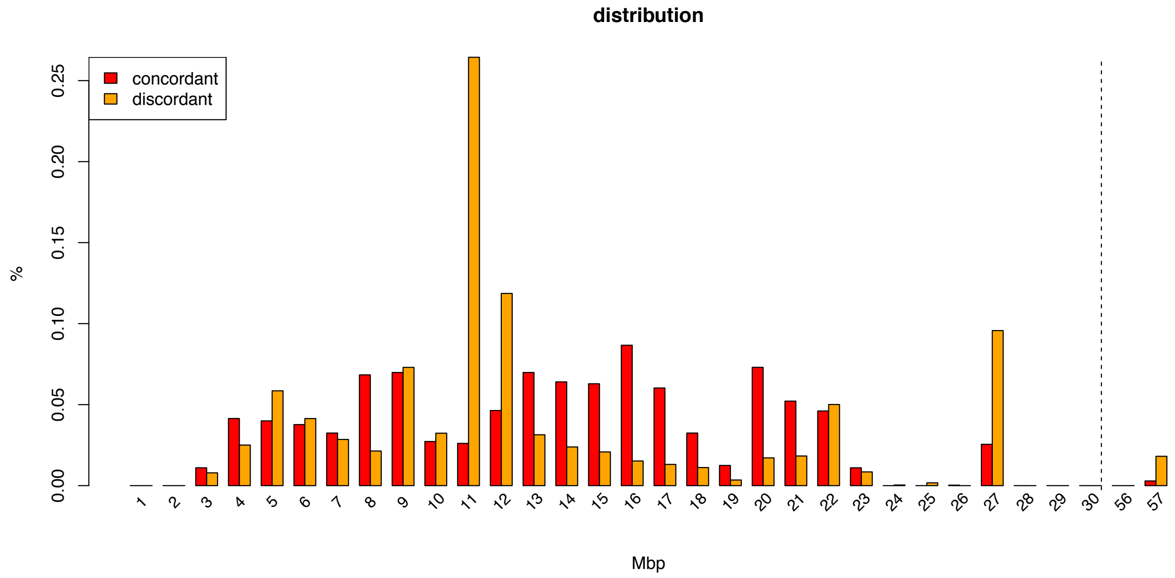

Supplementary Figure 11

Position of concordant and discordant genotypes as defined in the supplementary section ‘Genotype concordance to 1000 genomes calls’ using the Y chromosomal sequences from the publicly available sequence data from the 1000 genomes project (Poznik et al).

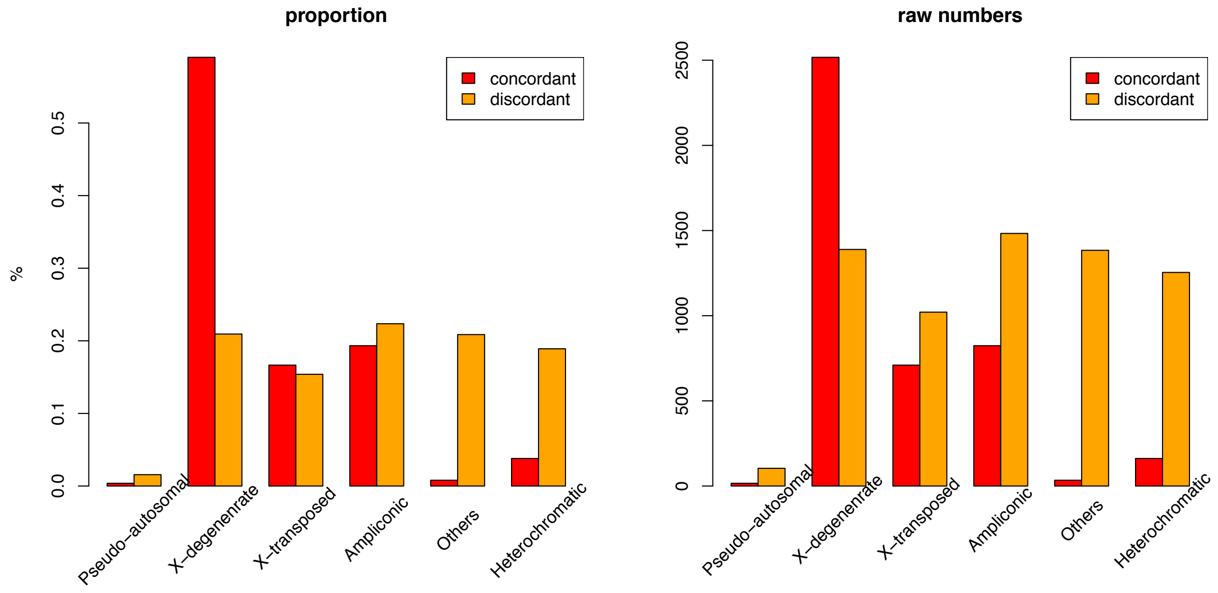

Supplementary Figure 12

Proportion and number of concordant and discordant genotypes as defined in the supplementary section 'Genotype concordance to 1000 genomes calls' using the Illumina data produced for this project, stratified by their respective sequence classes.

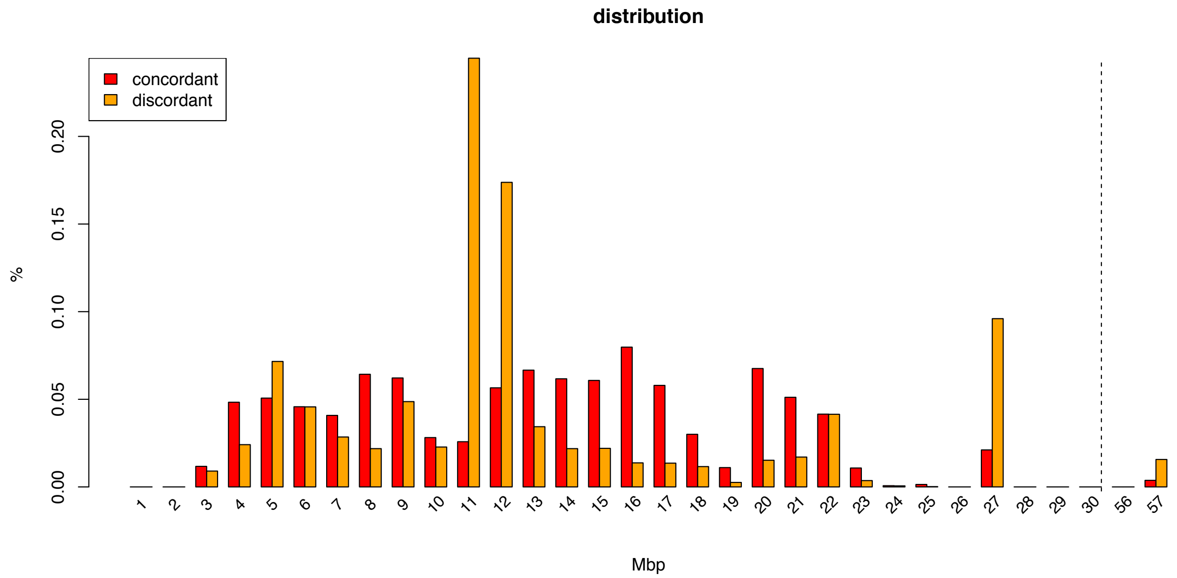

Supplementary Figure 13

Position of concordant and discordant genotypes as defined in the supplementary section ‘Genotype concordance to 1000 genomes calls’ using the Illumina data produced for this project.

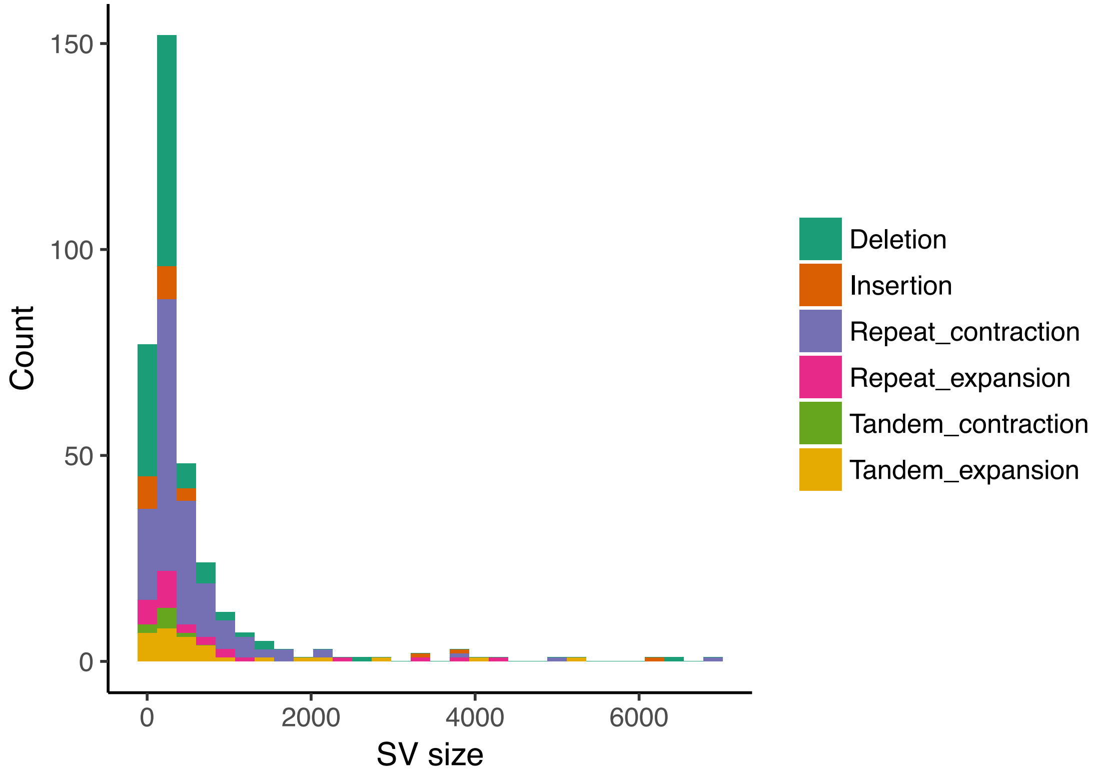

Supplementary Figure 14

Histogram of size distribution of SV calls

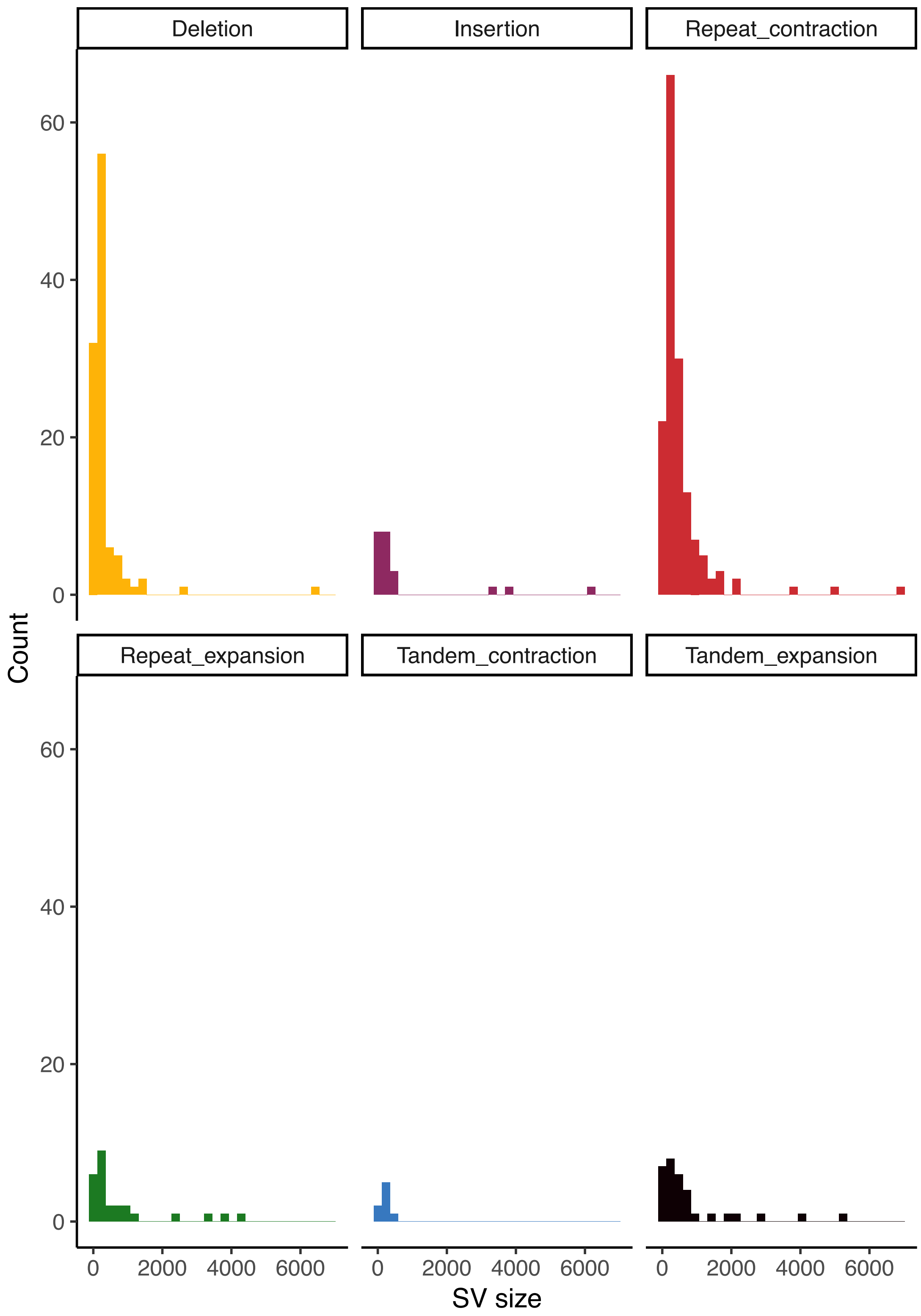

Supplementary Figure 15

Structural variant size distribution of variants of at least 50 bp, stratified by SV-types.

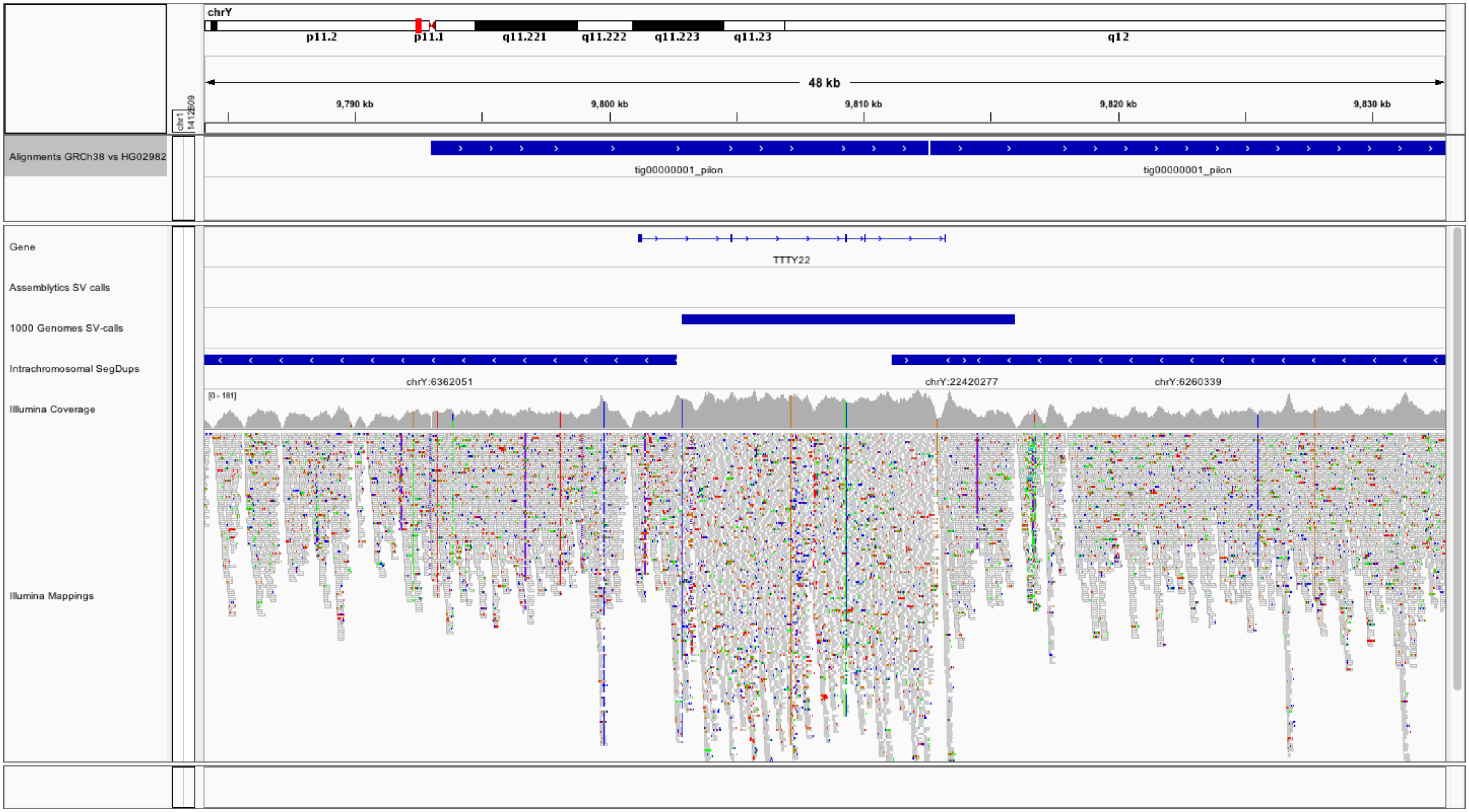

Supplementary Figure 16

IGV-screenshot 1000 genomes variant: Duplication at the TTTY22 locus confirmed with read-depth duplication detection

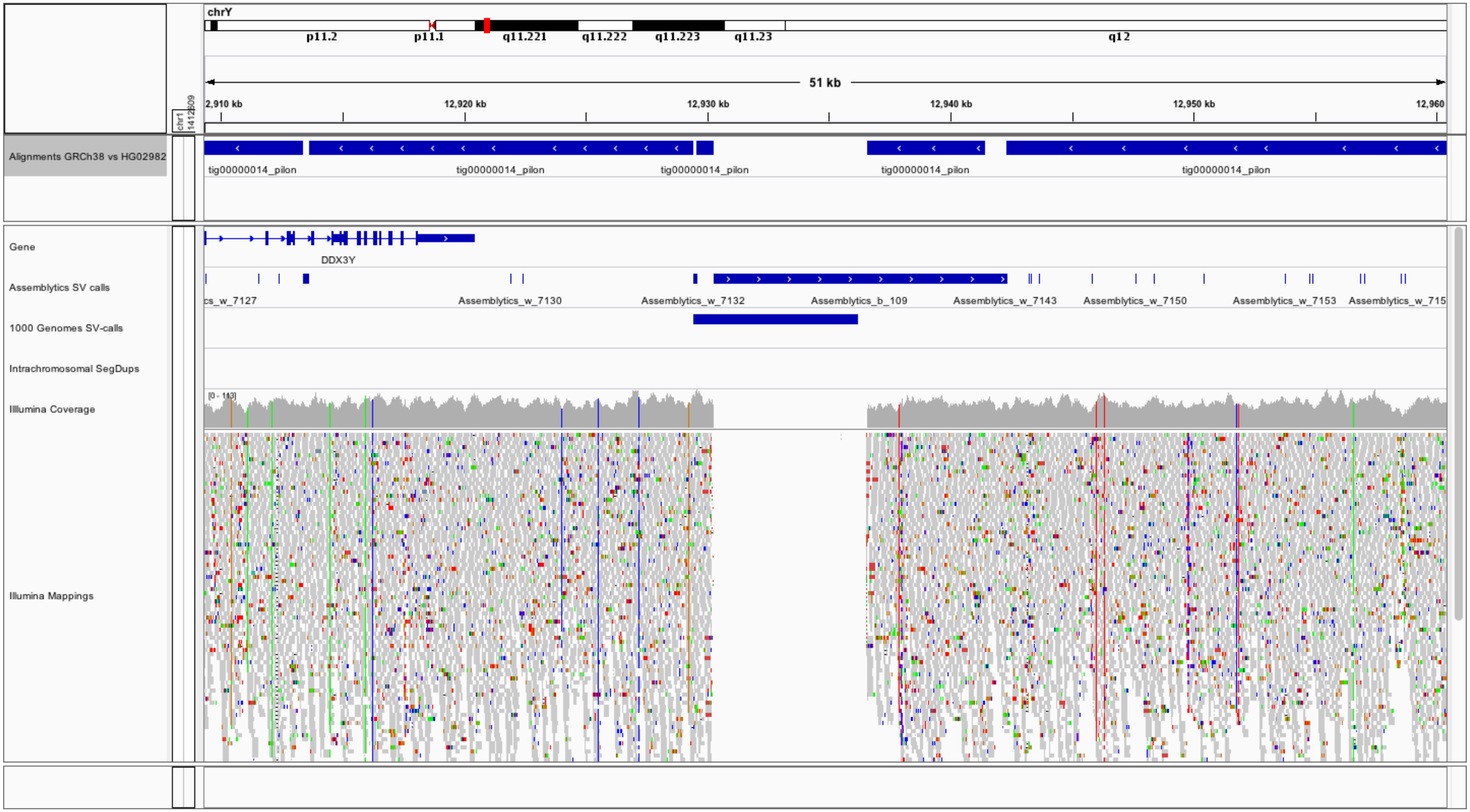

Supplementary Figure 17

IGV-screenshot 1000 genomes variant: Deletion from the reference nearby DDX3Y. The variant is contained within a SV call from Assemblytics with different boundaries.

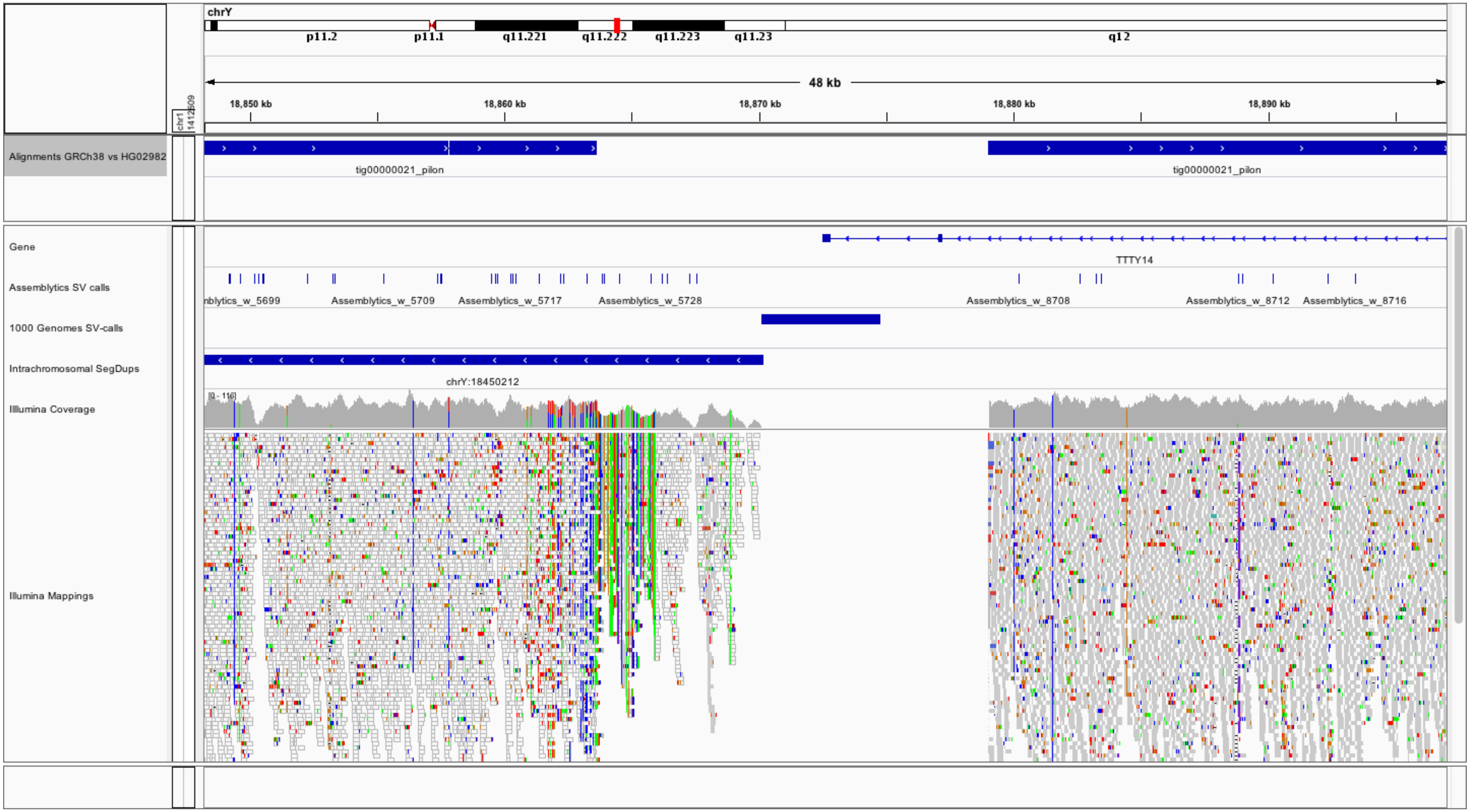

Supplementary Figure 18

IGV-screenshot 1000 genomes variant: Deletion nearby a segmental duplication. The HG02982 assembly enhances breakpoint resolution for the variant

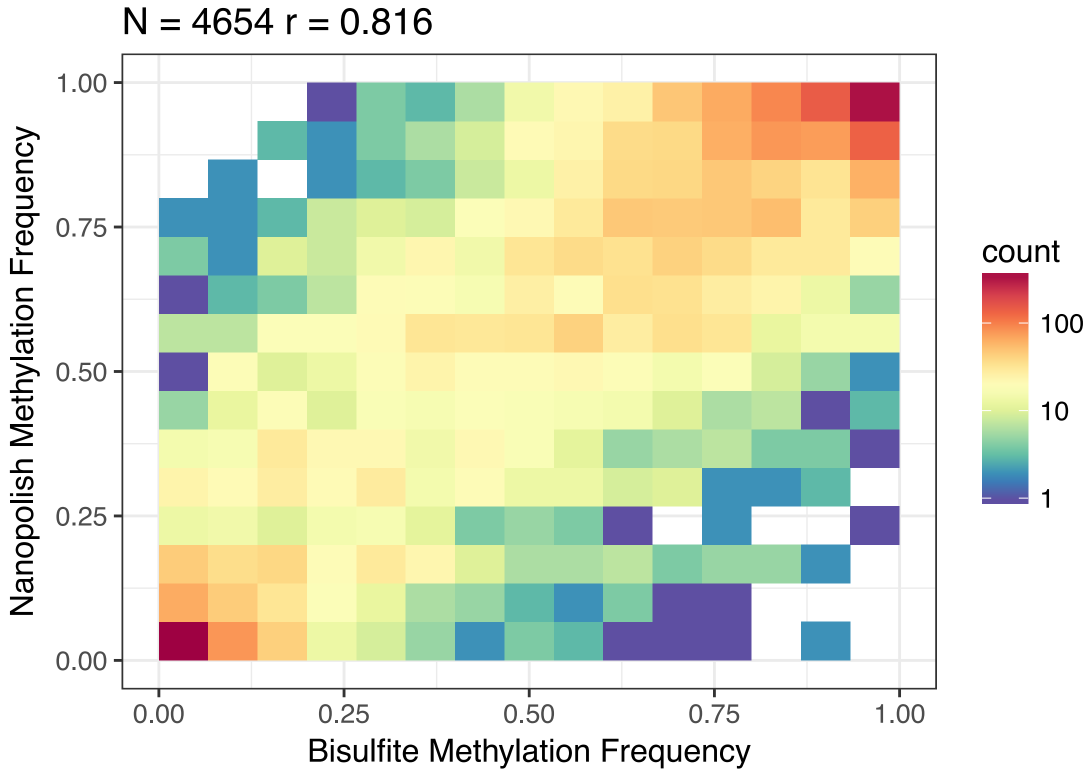

Supplementary Figure 19

Concordance of methylation frequency at CpG sitesbetween Illumina WGBS data and Nanopolish. Only sites with at least 10X in either dataset were included in the comparison (N=4654) to avoid potential biases due to differences in sequencing coverage. The call sets are correlated with a pearson's r of 0.816

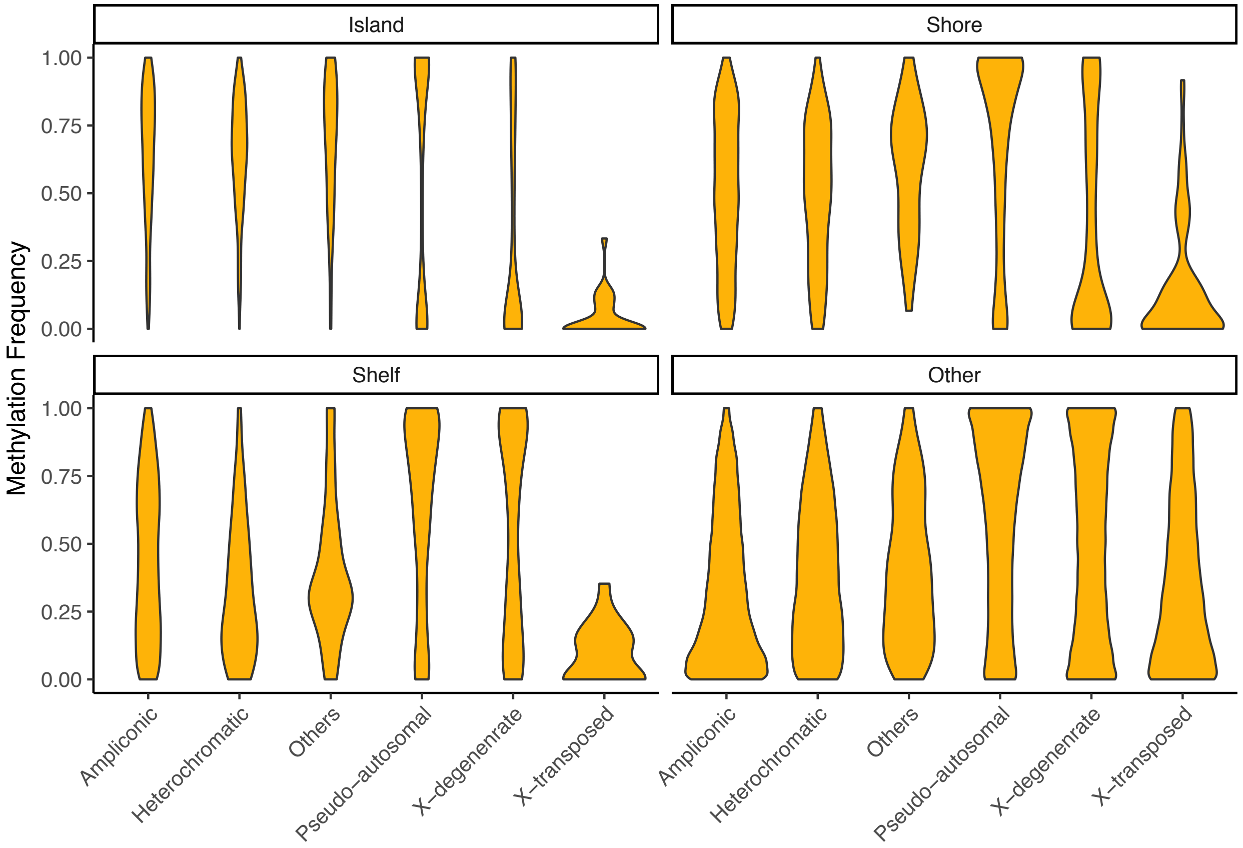

Supplementary Figure 20

Methylation frequency called from Nanopore data of CpG sites falling into CpG islands, CpG shores, CpG shelfs or all other CpG sites, stratified by different sequence classes. Only sites with coverage between 10-100X where included in the analysis.

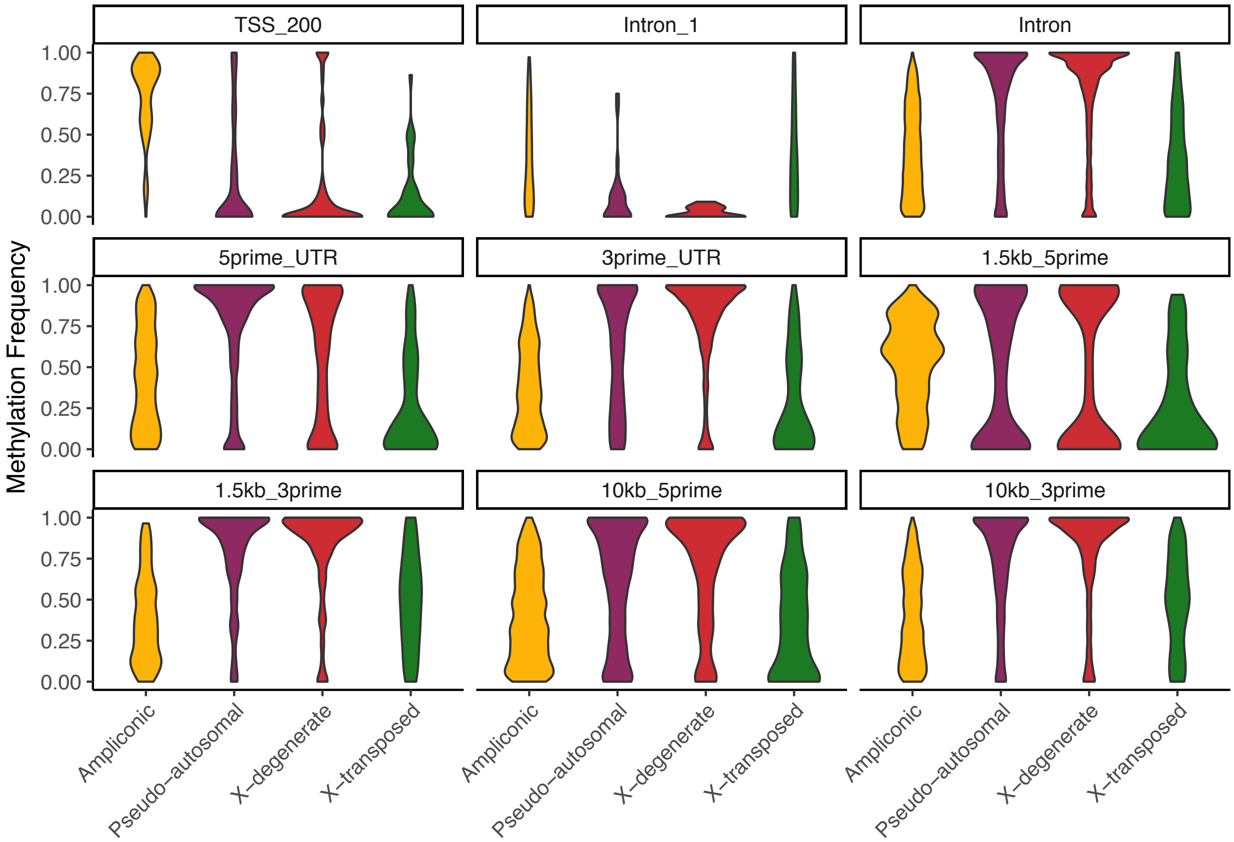

Supplementary Figure 21

Methylation frequency of CpG sites intersecting different region in or nearby protein coding genes, stratified by sequence classes. TSS methylation is different between the Ampliconic and all other sequence classes, suggesting differential regulation of genes within those regions. Only sites with coverage between 10-100X where included in the analysis.

Supplementary Tables

Supplementary Table 1

	First purification (ng/µl)	Second purification (ng/µl)
Qubit	15.5	28.8
NanoDrop	28.2	130.5

Quantification values of the DNA after purifications by dialysis and SPRI beads (see methods). Two rounds of purification with equal amount of input were performed. The quantification values are largely inconsistent between them. In either case Qubit (a method based on an intercalating dye) estimates a lower concentration then NanoDrop (a method based on the detection of aromatic groups). The quantification issues are consistent with the hypothesis that the intercalant used by qubit competes with the residual dyes (Hoechst and Chromomycin) that are not removed by dialysis, and this method therefore underestimates the amount of DNA. Conversely, Hoechst and Chromomycin contain aromatic groups that might lead to the overestimation of the concentration based on NanoDrop. We observed the concentration values of Qubit and NanoDrop to converge with time when performing dialysis (data not shown).

Supplementary Table 2

Run ID	Run name	number Reads	n50 (bp)	average length (bp)	median length (bp)	Yield (bp)
20170719_CGL_12_4345AA_CRGpc2_48H_FAE31760	run 1	123976	17303	7240	2931	897601496
20170724_CGL_12_4412AA_CRGpc2_48H_FAE31835	run 2	90126	16795	7284	3136	656556831
20170824_CGL_12_5300AA_CRGpc1_48H_FAH16306	run 3	75507	22341	8094	2462	611214526
20170824_CGL_12_5301AA_CRGpc2_48H_FAH12212	run 4	15919	23790	10286	4639	163751955
all		305528	18687	7623	2971	2329124808

Summary statistics of read lengths and yields for all runs.

Supplementary Table 3

**Size range**	**Type**	**Cumulative length**	**Number**
**10..50**	Deletion	8151	496
	Insertion	2861	163
	Repeat_contraction	376	11
	Repeat_expansion	129	4
	Tandem_expansion	92	2
**50..500**	Deletion	16518	94
	Insertion	3099	18
	Repeat_contraction	27117	109
	Repeat_expansion	3734	17
	Tandem_contraction	1484	8
	Tandem_expansion	4188	19
**500+**	Deletion	18099	12
	Insertion	13869	4
	Repeat_contraction	54765	44
	Repeat_expansion	18227	9
	Tandem_expansion	22514	13
**>10**	Deletion	42102	528
	Insertion	19667	167
	Repeat_contraction	82258	164
	Repeat_expansion	22090	30
	Tandem_contraction	1484	8
	Tandem_expansion	26794	34
**>50**	Deletion	34617	106
	Insertion	16968	22
	Repeat_contraction	81882	153
	Repeat_expansion	21961	26
	Tandem_contraction	1484	8
	Tandem_expansion	26702	32
	**Total**	**183614**	**347**

SV-calls by Assemblytics stratified by size. There is a clear deletion bias across all size ranges.

Supplementary Table 4

Matches	Mismatches	Insertions	Deletions	perc. Identitiy	perc. Identitiy SNP only	assembly
20,242,759	30,248	12,633	202,425	98.80	99.85	NP_variants_min_0
20,243,039	30,301	12,701	202,508	98.80	99.85	NP_variants_min_0.1
20,234,195	30,500	12,886	203,878	98.79	99.85	NP_variants_min_0.2
20,206,603	30,969	13,277	208,557	98.76	99.85	NP_variants_min_0.3
20,251,615	31,840	13,838	219,506	98.71	99.84	NP_variants_min_0.4
20,198,119	32,856	14,191	238,764	98.60	99.84	NP_variants_min_0.5
20,036,473	33,449	14,533	263,428	98.47	99.83	NP_variants_min_0.6
19,969,862	34,413	14,867	288,018	98.34	99.83	NP_variants_min_0.7
19,944,500	35,090	15,062	299,929	98.28	99.82	NP_variants_min_0.8
19,908,562	35,372	15,195	304,139	98.25	99.82	NP_variants_min_0.9
20,125,302	29,331	13,094	188,110	98.87	99.85	NP_consensus
20,459,952	24,716	15,154	27,771	99.67	99.88	NP_consensus_pilon
20,602,540	50,440	8,321	20,051	99.62	99.76	NP_consensus_pilon_racon

Comparison of different polishing approaches and their effects on the assembly. The assemblies were polished using nanopolishes variant calling module and inserting the resulting variants, at varying rates of support-fraction cut-offs (i.e. the proportion of reads that support a given variant, assemblies NP_variants_min_X). We also include polishing using nanopolishes consensus module (NP_consensus), Nanopolish consensus+pilon (NP_consensus_pilon) and Nanopolish consensus+pilon+racon NP_consensus_pilon_racon. Overall, the Nanopolish consensus based polishing produces higher ab initio identities than the 'variant calling' polishing. Additionally, using pilon after the consensus polishing gets rid of most remaining deletion errors in the assembly. While adding one round of racon polishing slightly lowers the identity, it polishes about half the remaining insertion errors, and adds ~140Kb of matches to the alignment, despite also doubling the number of mismatches.

Supplementary Table 5

Minimum Coverage	Calls Nanopore	Calls Illumina WGBS	Percentage of all CpG Nanopore	Percentage of all CpG Illumina WGBS
1	172,278	84,610	76.26	37.45
5	156,204	38,903	69.14	17.22
10	121,392	5,818	53.73	2.58

Number of CpG that were addressed in either the Nanopore methylation calls, or the Illumina WGBS calls. There is a substantial difference is sequencing depth between the two callsets (30X Nanopore, and around 5X Illumina WGBS on the Y chromosome), however, there are regions on the Y chromosome that are not accessible to either short reads or whole genome shotgun sequencing because of mapping ambiguities, namely the Pseudo-autosomal, the X-transposed region and to some degree the Ampliconic regions. There are a total of 225,912 CpG sites on the Y chromosome.

Supplementary References

1. Poznik, G. D. *et al.* Punctuated bursts in human male demography inferred from 1,244 worldwide Y-chromosome sequences. *Nat. Genet.* **48,** 593–599 (2016).

2. Dutheil, J. Y., Gaillard, S. & Stukenbrock, E. H. MafFilter: A highly flexible and extensible multiple genome alignment files processor. *BMC Genomics* **15,** (2014).

3. Lawrence, M., Gentleman, R. & Carey, V. rtracklayer: An R package for interfacing with genome browsers. *Bioinformatics* **25,** 1841–1842 (2009).

4. Lawrence, M. *et al.* Software for Computing and Annotating Genomic Ranges. *PLoS Comput. Biol.* **9,** 1–10 (2013).

5. Li, H. & Durbin, R. Fast and accurate short read alignment with Burrows-Wheeler transform. *Bioinformatics* **25,** 1754–1760 (2009).

6. Li, H. *et al.* The Sequence Alignment/Map format and SAMtools. *Bioinformatics* **25,** 2078–2079 (2009).

7. McKenna, A. *et al.* The Genome Analysis Toolkit: A MapReduce framework for analyzing next-generation DNA sequencing data. *Genome Res.* **20,** 1297–1303 (2010).
