# Supplementary material for: Selective single molecule sequencing and assembly of a human Y chromosome of African origin
Source: Nat Commun. 2019 Jan 2;10:4. doi: 10.1038/s41467-018-07885-5 (PMC6315018; doi:10.1038/s41467-018-07885-5)
Supplement: Supplementary file 3 — Description of Additional Supplementary Files [file 41467_2018_7885_MOESM3_ESM.pdf]

Description of Additional Supplementary Files

Supplementary Data 1:	Enrichment specificity nanopore data
Supplementary Data 2:	Enrichment specificity illumina data
Supplementary Data 3:	Sequence class coordinates of GRCh38 chrY
Supplementary Data 4:	Gene family resolution
Supplementary Data 5:	Gene copy number based on read depth
Supplementary Data 6:	NA24385 assembly contig ID of mappings to chrY
