# Supplementary material for: Selective single molecule sequencing and assembly of a human Y chromosome of African origin
Source: Nat Commun. 2019 Jan 2;10:4. doi: 10.1038/s41467-018-07885-5 (PMC6315018; doi:10.1038/s41467-018-07885-5)
Supplement: Supplementary file 11 — Reporting Summary [file 41467_2018_7885_MOESM11_ESM.pdf]

nature research

Corresponding author(s): Lukas F.K. Kuderna
Esther Lizano
Tomas Marques-Bonet

Reporting Summary

Nature Research wishes to improve the reproducibility of the work that we publish. This form provides structure for consistency and transparency in reporting. For further information on Nature Research policies, see Authors & Referees and the Editorial Policy Checklist.

Statistical parameters

When statistical analyses are reported, confirm that the following items are present in the relevant location (e.g. figure legend, table legend, main text, or Methods section).

n/a	Confirmed	
☐	☒	The exact sample size (*n*) for each experimental group/condition, given as a discrete number and unit of measurement
☐	☒	An indication of whether measurements were taken from distinct samples or whether the same sample was measured repeatedly
☐	☒	The statistical test(s) used AND whether they are one- or two-sided<br>*Only common tests should be described solely by name; describe more complex techniques in the Methods section.*
☐	☒	A description of all covariates tested
☒	☐	A description of any assumptions or corrections, such as tests of normality and adjustment for multiple comparisons
☐	☒	A full description of the statistics including central tendency (e.g. means) or other basic estimates (e.g. regression coefficient) AND variation (e.g. standard deviation) or associated estimates of uncertainty (e.g. confidence intervals)
☒	☐	For null hypothesis testing, the test statistic (e.g. *F*, *t*, *r*) with confidence intervals, effect sizes, degrees of freedom and *P* value noted<br>*Give P values as exact values whenever suitable.*
☒	☐	For Bayesian analysis, information on the choice of priors and Markov chain Monte Carlo settings
☒	☐	For hierarchical and complex designs, identification of the appropriate level for tests and full reporting of outcomes
☐	☒	Estimates of effect sizes (e.g. Cohen's *d*, Pearson's *r*), indicating how they were calculated
☒	☐	Clearly defined error bars<br>*State explicitly what error bars represent (e.g. SD, SE, CI)*

Our web collection on statistics for biologists may be useful.

Software and code

Policy information about availability of computer code

Data collection	A description of all software used is provided in the manuscript.<br>Software used for data collection:<br>MinKNOW Mk1B v1.7.10 (Oxford Nanopore)<br>Casava (illumina Inc)
Data analysis	A description of all software used is provided in the manuscript.<br>Software used for data analysis<br>Albacore 1.1<br>Albacore 2.1<br>Canu v. 1.6<br>Nanopolish v. 0.8.4<br>bwa mem v. 0.7.120<br>trimgalore v 3.7<br>Picardtools v. 2.8.2<br>Pilon v. 1.22<br>racon v. 1.3.1<br>GATK v. 4.0.0.0<br>RepeatMasker v. 4.0.7<br>rmblastn v. 2.6.0+

April 2018

last v. 914
Mummer v. 3.22
Assemblytics (https://github.com/MariaNattestad/assemblytics, 2857b87599c15cc99fea291c1c5672c0f22c46d9)
GEM v 2
mrCanavar v. 0.51
GMAP v. 20170317
exonerate v. 2.2.0
gemBS v 3.2.1

For manuscripts utilizing custom algorithms or software that are central to the research but not yet described in published literature, software must be made available to editors/reviewers upon request. We strongly encourage code deposition in a community repository (e.g. GitHub). See the Nature Research guidelines for submitting code & software for further information.

Data

Policy information about availability of data

All manuscripts must include a data availability statement. This statement should provide the following information, where applicable:

- Accession codes, unique identifiers, or web links for publicly available datasets
- A list of figures that have associated raw data
- A description of any restrictions on data availability

All raw sequencing data for this study has been deposited at the European Nucleotide Archive (ENA) under the study accession 'PRJEB28143[https://www.ebi.ac.uk/ena/data/view/PRJEB28143]'. The assembly is deposited at the ENA under the accession 'ULGL01000000[https://www.ebi.ac.uk/ena/data/view/ULGL01000000]'. The whole genome shotgun assembly for the NA24385 individual, the repeat masker tracks for the GRCh38 chrY and HG02982 assemblies, and the methylation calls from the Illumina WGBS and the Nanopore data are deposited at Figshare under the DOI '10.6084/m9.figshare.7358480[https://doi.org/10.6084/m9.figshare.7358480.v1]'. The source data underlying Figures 1a-d and 2a-c are provided as a Source Data File. A reporting summary for this Article is available as a Supplementary Information file.
All other relevant data is available upon request.

Field-specific reporting

Please select the best fit for your research. If you are not sure, read the appropriate sections before making your selection.

☒ Life sciences ☐ Behavioural & social sciences ☐ Ecological, evolutionary & environmental sciences

For a reference copy of the document with all sections, see nature.com/authors/policies/ReportingSummary-flat.pdf

Life sciences study design

All studies must disclose on these points even when the disclosure is negative.

Sample size	We used a single sample for our study
Data exclusions	No data was exluded
Replication	We sequenced a single sample for our study, replication was not required
Randomization	We sequenced a single sample for our study, randomization was not required
Blinding	No blinding was required for this study

Reporting for specific materials, systems and methods

Materials & experimental systems

n/a	Involved in the study	
☐	☒	Unique biological materials
☒	☐	Antibodies
☐	☒	Eukaryotic cell lines
☒	☐	Palaeontology
☒	☐	Animals and other organisms
☒	☐	Human research participants

Methods

n/a	Involved in the study	
☒	☐	ChIP-seq
☐	☒	Flow cytometry
☒	☐	MRI-based neuroimaging

Unique biological materials

Policy information about availability of materials

Obtaining unique materials	This study used the human lymphoblastoid cell line HG02982. The cell line is supplied by and available through Coriell

Eukaryotic cell lines

Policy information about cell lines

Cell line source(s)	This study used the human lymphoblastoid cell line HG02982. The cell line is supplied by and available through Coriell
Authentication	The cell lines are authenticated at the source (Coriell), see https://www.coriell.org/0/pdf/CC_Process_Flow.pdf . The cell line was sequenced.
Mycoplasma contamination	Cell lines were not tested for mycoplasma contamination, but contamination would be detectable though genome sequence analysis
Commonly misidentified lines (See ICLAC register)	No commonly misidentified lines were used

Flow Cytometry

Plots

Confirm that:

- ☒ The axis labels state the marker and fluorochrome used (e.g. CD4-FITC).
- ☒ The axis scales are clearly visible. Include numbers along axes only for bottom left plot of group (a 'group' is an analysis of identical markers).
- ☒ All plots are contour plots with outliers or pseudocolor plots.
- ☒ A numerical value for number of cells or percentage (with statistics) is provided.

Methodology

Sample preparation	Detailed sample preparation steps are provided in the methods section of the manuscript:<br>Briefly, lymphoblastoid cell lines were cultured in RPMI 1640 medium supplemented with 2mM L-glutamine (Invitrogen, ref. 21875-034), 15% fetal bovine serum and antibiotics (Penicillin and Streptomycin (Invitrogen, ref. 15140-122)) at initial concentration no less than 150,000 viable cells/ml. Near confluence, cells were subcultured to 50%. After 24h, the cells were blocked in mitosis by adding Colcemid to the culture (10 µg/ml demecolcine solution (Gibco, ref. 15210-040)) to a final concentration of 0.1 µg/ml and incubated for an additional 6-7 h. To swell and stabilize mitotic cells, they were centrifuged 5 min at 300×g at room temperature. The pellet was slowly resuspended in 10 mL hypotonic solution (Hypotonic solution: 75 mM KCl, 10 mM MgSO4, 0.2 mM spermine, 0.5 mM spermidine. pH 8.0), incubated for 10 min at room temperature. After the incubation in the hypotonic solution, the swollen cells were centrifuged at 300×g for 5 min. The cell pellet was resuspended in 1.5 mL of ice-cold polyamine isolation buffer (PAB: 15 mM Tris, 2 mM EDTA, 0.5 mM EGTA, 80 mM KCl, 3 mM dithiothreitol, 0.25% Triton X-100, 0.2 mM spermine, 0.5 mM spermidine. pH 8.0) for 20 min to release the chromosomes.<br>To ensure the integrity of the chromosomes, their morphology was checked before staining them. To this end, the pellet was vigorously vortexed for 30 s to liberate the chromosomes from the mitotic cells. The suspension was filtered through a 35 µm mesh filter and stored at 4°C until its sorting. Finally, chromosomes were stained with chromomycin-A3 (Sigma, ref. C2659) and Hoechst 33258 (Invitrogen, ref. H3569) at a final concentration of 40 µg/ml and 5 µg/ml respectively in presence of divalent cations (10 mM MgSO4 (Sigma, ref. 60142)). Staining was performed for at least 8 h at 4°C, to allow the dyes to equilibrate.<br>Before the sample analysis on a cell sorter, potassium citrate was added to a final concentration of 10 mM (Sigma, ref. 89306) to enhance peak resolution in the flow karyotype.
Instrument	BD Influx cell sorter (Becton Dickinson, San Jose, CA)
Software	BD FACSTM Software (v. 1.0.0.0.650, Becton Dickinson, San Jose, CA).
Cell population abundance	Cell population abundance is not applicable, as we are sorting chromosome. We find Y chromosomal sequenced to be 110-fold enriched compared to sampling a form a full human genome.
Gating strategy	Just one gate to sort Y-Chromosome was made on standard Hoechst vs. chromomycin-A3 bivariate flow karyogram (see Fig. 1A). Consequently, no sequential gating was applied for Y-Chromosomes sorting.

☒ Tick this box to confirm that a figure exemplifying the gating strategy is provided in the Supplementary Information.
